# Oral administration of pioglitazone inhibits pulmonary hypertension by regulating the gut microbiome and plasma metabolome in male rats

**DOI:** 10.14814/phy2.70174

**Published:** 2024-12-31

**Authors:** Zizhou Zhang, Yaru Liang, Shaocong Mo, Mingming Zhao, Yi Li, Chenting Zhang, Xiaoqian Shan, Shiyun Liu, Jing Liao, Xiaoyun Luo, Junqi Zhu, Chen Wang, Qian Jiang, Chi Hou, Wei Hong, Ning Lai, Yuqin Chen, Lei Xu, Wenju Lu, Jian Wang, Zhongfang Wang, Kai Yang

**Affiliations:** ^1^ Department of Laboratory Medicine The Affiliated Qingyuan Hospital (Qingyuan People's Hospital), Guangzhou Medical University Qingyuan Guangdong China; ^2^ State Key Laboratory of Respiratory Disease, Department of Pulmonary and Critical Care Medicine, The First Affiliated Hospital Guangzhou Medical University Guangzhou Guangdong China; ^3^ National Clinical Research Center for Respiratory Disease Guangzhou Institute of Respiratory Health, Guangzhou Medical University Guangzhou Guangdong China; ^4^ Department of Pulmonary and Critical Care Medicine The Affiliated Hospital of Inner Mongolia Medical University Hohhot China; ^5^ Department of Pulmonary Diseases The Third Affiliated Hospital of Sun Yat‐Sen University, Institute of Respiratory Diseases of Sun Yat‐Sen University Guangzhou Guangdong China; ^6^ School of Medicine Southern University of Science and Technology Shenzhen Guangdong China; ^7^ Guangzhou National Laboratory Guangzhou International Bio Island Guangzhou Guangdong China; ^8^ Department of Neurology, Guangzhou Women and Children's Medical Center Guangzhou Medical University Guangzhou Guangdong China; ^9^ GMU‐GIBH Joint School of Life Sciences Guangzhou Medical University Guangzhou Guangdong China

**Keywords:** gut microbiome, peroxisome proliferator‐activated receptor gamma, pioglitazone, plasma metabolome, pulmonary hypertension

## Abstract

The oral administrated thiazolidinediones (TZDs) have been widely reported to alleviate experimental pulmonary hypertension (PH). However, previous studies mainly focused on their beneficial effects on the cardiopulmonary vascular system but failed to determine their potential roles on gut microenvironment. This study aims to investigate the effects of pioglitazone, an oral TZD drug, on gut microbiome in classic PH rat models induced by hypoxia (HPH) or SU5416/hypoxia (SuHx‐PH) and evaluate the therapeutic potential of supplementation of selective probiotics for experimental PH. Pioglitazone remarkably inhibited the PH pathogenesis in both models and reshaped the gut microbiome and plasma metabolome. Correlation analyses represented strong and unique association between the protective metabolites and bacteria genera (*Roseburia*, *Lactobacillus*, and *Streptococcus*) that were positively stimulated by pioglitazone. Supplementation of selective probiotics *Roseburia intestinalis* (*R. intestinalis*) partially attenuated SuHx‐PH and rebuilt a novel gut microbiome and host metabolome. This study reports for the first time that oral administration of pioglitazone protects PH by regulating the gut microbiome and host metabolome, providing novel insights for the TZD drugs. The data also supports that modulation of gut microbiota by supplementation of selective probiotics could be a novel effective therapeutic strategy for the treatment of PH.

## Graphical abstract

Treatment of pioglitazone protects experimental pulmonary hypertension (PH) by reshaping the gut microbiome and plasma metabolome, enhancing specific gut bacteria (*Roseburia*, *Lactobacillus* and *Streptococcus*) and plasma metabolites (Clusters 1, 3, and 5). Transplantation of the probiotic bacteria, *Roseburia intestinalis*, partially attenuates PH and rebuilds the gut microbiome and plasma metabolome.

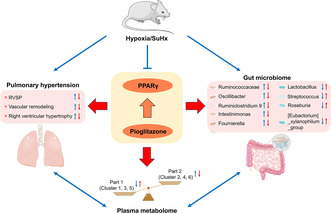

## 1 INTRODUCTION

The oral administrated thiazolidinedione (TZD) class of peroxisome proliferator‐activated receptor gamma (PPARγ) ligands, such as pioglitazone and rosiglitazone, are FDA‐approved oral drugs for the treatment of type 2 diabetes, which have been proven as promising reagents for the treatment of pulmonary hypertension (PH) (Hansmann et al., 2020). However, previous studies mainly focused on their beneficial effects on the cardiopulmonary vascular system but failed to determine their potential roles on gut microenvironments. PPARγ belongs to the PPARs family of nuclear receptors, which regulate gene transcription of pathways including metabolism and inflammation (Tseng et al., 2019). During the PH development, altered metabolism involves a switch to glycolysis, fatty acid oxidation, and production of reactive oxygen species (D'Alessandro et al., 2018). Legchenko et al. reported that activation of PPARγ using pioglitazone can reverse the metabolic changes that occur in PH, likely through the epigenetic and transcriptional regulation relating to the disturbed lipid metabolism and mitochondrial morphology and function (Legchenko et al., 2018). The detailed molecular mechanisms underlying the therapeutic effects of pioglitazone especially its role on the gut microenvironments deserve further evaluation.

Studies have linked the relationship between gut microbiome and metabolic homeostasis (Kamada et al., 2013). The disease‐associated changes in gut microbiota composition are referred to as dysbiosis. The impaired bacterial ecosystem may be therapeutically targeted by probiotics—live strains of selected bacteria—or prebiotics‐food components (Kristensen et al., 2016). Multiple cardiovascular, metabolic, and respiratory diseases, such as hypertension and heart failure, have been linked to gut dysbiosis (Velmurugan et al., 2020), which is normally characterized by a less diverse and less rich microbial composition (Yang et al., 2015). Moreover, meta‐analysis of the human studies supports the idea that supplementation with probiotics restores the proper gut microbiota and improves disease biomarkers (Robles‐Vera et al., 2017). As previously reviewed, the host microbiota plays key role to induce the metabolic and immunopathogenic shifts, together contribute to the disease development of PH (Koch et al., 2017). In fact, emerging studies have documented the correlation between altered microbiota profile and disease parameters of PH, including the altered gut microbiota (Callejo et al., 2018; Sanada et al., 2020; Sharma, Oliveira, Yang, Karas, et al., 2020; Sharma, Oliveira, Yang, Kim, et al., 2020) and airway microbiota (Zhang et al., 2020) in patients with PH or experimental PH animal models.

In this study, we demonstrated the effects of oral administrated pioglitazone on the profile changes of gut microbiome and plasma metabolome in PH rat models induced by hypoxia (HPH) or SU5416/hypoxia (SuHx‐PH). The gut microbiota communicates with distal organs by producing numerous metabolites that may be absorbed into the systemic circulation and exert biological effects (The Integrative Human Microbiome Project, 2014). Using non‐biased correlation analysis, this study uncovered unique significant correlation signature between selective gut microbiome and plasma metabolome, strongly implying that treatment with pioglitazone can inhibit established PH, at least partially, by inducing metabolic shifts, retrieving the gut dysbiosis, and reshaping the plasma metabolome. Moreover, supplementation of specific enriched probiotic bacteria *Roseburia intestinalis* (*R. intestinalis*) partially attenuated SuHx‐PH, providing essential evidence that a microbiota‐driven strategy could modulate the PH pathogenesis, leading a new avenue for future mechanistic and translational research for PH.

## 2 MATERIALS AND METHODS

### 2.1 Animal procedure

The animal protocols were approved and conducted by the Animal Care and Use Committee of the First Affiliated Hospital of Guangzhou Medical University (ethical approval number: 2018–456). All animal studies conformed to the ARRIVE guidelines. Adult male Sprague Daley (SD) rats (weighing 200 ~ 250 g) were purchased from the Guangdong Provincial Medical Experimental Animal Centre, fed with AIN‐93 M purified diets (#XT93M, Jiangsu Xietong Pharmaceutical Bio‐engineering Co., Ltd., Nanjing, China), and housed in the Specific Pathogen Free (SPF) grade animal room of the State Key Laboratory of Respiratory Disease, the First Affiliated Hospital of Guangzhou Medical University. Rats were randomly divided into different experimental groups. For the SuHx groups, rats were injected with 20 mg/kg of SU5416 (#HY‐10374, MedChemExpress, USA) dissolved in DMSO and subsequently exposed to chronic hypoxia (10% O_2_) for 3 weeks, followed by 3 weeks of normoxic exposure with or without treatment of pioglitazone (20 mg/kg/day, orally, #6939863707099, WANBANG Biopharmaceuticals, Xuzhou, China). After treatments, the rats were anesthetized with sodium pentobarbital (50 mg/kg). The fecal and blood samples were collected for analysis of gut microbiome and plasma metabolome, respectively. The rats were overdose anesthetized and the heart and lungs were isolated for further experiments. The rats were euthanized due to exsanguinations.

### 2.2 Treatment of antibiotics and Roseburia intestinalis (*R. intestinalis*)

For the microbial depletion by antibiotics, rats were treated with the reported antibiotics cocktail including ampicillin, vancomycin, metronidazole, and neomycin in drinking water for 8 days. The antibiotic cocktail was dissolved in sterile water containing ampicillin (110 mg/kg, #69–52‐3, Meilunbio, Shanghai, China), vancomycin (55 mg/kg, #1404‐93‐9, Meilunbio), metronidazole (110 mg/kg, #443–48‐1, Meilunbio), and neomycin (110 mg/kg, #1405‐10‐3, Meilunbio). Antibiotics were removed, and the rats were subjected to hypoxic exposure for 21 days with or without supplementation of *R. intestinalis* (2 × 10^8^ CFUs, oral gavage) for 8 days. *R. intestinalis* DSM 14610 was purchased from DSMZ (Deutsche Sammlung von Mikroorganismen und Zellkulturen), validated by Sangon Biotech (Shanghai) Co., Ltd., amplified, and extracted according to the manufacturer's instructions. *R. intestinalis* was stored and diluted in sterile anaerobic 1 × PBS per serving. The strain of *R. intestinalis* was validated by sequencing.

### 2.3 Hemodynamic and histological measurements

The right ventricular pressure was recorded using a 23‐gauge needle filled with 0.3% heparinized saline connected to a pressure transducer and a BIOPAC MP150 data acquisition system (BIOPAC Systems, Inc., Santa Barbara, USA). The fluid‐filled needle was inserted into the right ventricle (RV) via the diaphragm. The right ventricular hypertrophy was indexed by Fulton Index, which represents the weight ratio of RV to left ventricle (LV) + septum (S) (RV/[LV + S]). The lungs were flushed by 5 mL of saline to wash out the residual blood. Then, the lung lobes were fixed with 4% paraformaldehyde under a constant pressure of 20 cm H_2_O, embedded in paraffin, and sectioned at 5 μm thickness. Then, the lung slides from the five groups of rats were used for hematoxylin and eosin (H&E) staining or immunofluorescence staining with antibodies of smooth muscle‐specific marker α‐SMA (1:200, #A5228, Sigma‐Aldrich) and endothelial‐specific marker vWF (1:200, #F3520, Sigma‐Aldrich), and then with secondary antibodies against mouse (Alexa Fluor® 488, #ab150113, abcam) and rabbit (Alexa Fluor® 594, #ab150080, abcam), respectively. The nuclei were labeled by DAPI (Invitrogen). The immunofluorescent intensity of α‐SMA and vWF was quantified by Image‐Pro Plus 6.0.

### 2.4 Echocardiographic assessments

The rats were anesthetized by inhaled isoflurane (1.5%) through a facemask, and the echocardiographic assessments were performed on a VisualSonics Vevo 2100 system (VisualSonics Inc., Toronto, ON, Canada) and a transducer (MS‐400, 20–46 MHz). The right heart function was reflected by specific indexes including the ratio of pulmonary acceleration time (PAT) to pulmonary ejection time (PET), right ventricular end‐systolic wall thickness (RVESWT), and right ventricular end‐diastolic wall thickness (RVEDWT).

### 2.5 Fecal sample DNA extraction, 16S rRNA sequencing, and data analysis

Fecal samples were collected, and DNA was extracted and quantified following the manufacturer's instructions. The V3‐V4 hypervariable regions of the bacteria 16S rRNA were amplified by using the primer pairs: 338F (5′‐ACTCCTACGGGAGGCAGCAG‐3′) and 806R (5′‐GGACTACHVGGGTWTCTAAT‐3′) by thermocycler PCR system (ABI GeneAmp 9700, ABI, USA), processed, and paired‐end sequenced (2 × 300) on an Illumina MiSeq platform (Illumina, San Diego, USA) according to the standard protocols by Majorbio Bio‐Pharm Technology Co. Ltd. (Shanghai, China). The taxonomy of each 16S rRNA gene sequence was analyzed by RDP Classifier algorithm (http://rdp.cme.msu.edu/) against the Silva (SSU123) 16S rRNA database using a confidence threshold of 70%.

### 2.6 Analysis of plasma metabolome

Blood samples (3 mL) were collected by anticoagulation, and plasma was obtained following the standard protocol of Novogene (Beijing, China). The samples were analyzed by LC–MS/MS on a Vanquish UHPLC system (Thermo Fisher) coupled with an Orbitrap Q Exactive series mass spectrometer (Thermo Fisher). The raw data files were processed using the Compound Discoverer 3.1 (Thermo Fisher) and matched with the mzCloud (https://www.mzcloud.org/), mzVault, and MassList database to obtain the accurate qualitative and relative quantitative results. Statistical analyses were performed using R (R version R‐3.4.3), Python (Python 2.7.6 version), and CentOS (CentOS release 6.6).

### 2.7 Statistical analysis

GraphPad Prism 7 and SPSS (version 23.0) were used to perform statistical analyses. Data were tested for normal distribution (Kolmgorov‐Smirnov test) and/or homogeneity of variance (Bartlett's test) by using SPSS and differences for ≥3 groups difference were assessed by one‐ or two‐way analysis of variance (ANOVA) followed by Bonferroni's multiple comparison test or Fishers LSD post hoc test. A *p* < 0.05 was considered statistically significant by one or two‐way analysis of variance (ANOVA) followed by Bonferroni's multiple comparison test or Fishers LSD post hoc test. Statistical analyses for the gut microbiome and plasma metabolome were performed based on the Majorbio Cloud Platform developed and maintained by the Shanghai Majorbio Bio‐Pharm Technology Co., Ltd., Shanghai, China (https://cloud.majorbio.com). The k‐means clustering analysis of plasma metabolome was performed by MathWorks MATLAB R2020a.

## 3 RESULTS

### 3.1 Pioglitazone inhibits experimental PH rat models induced by hypoxia or SuHx

**FIGURE 1:**
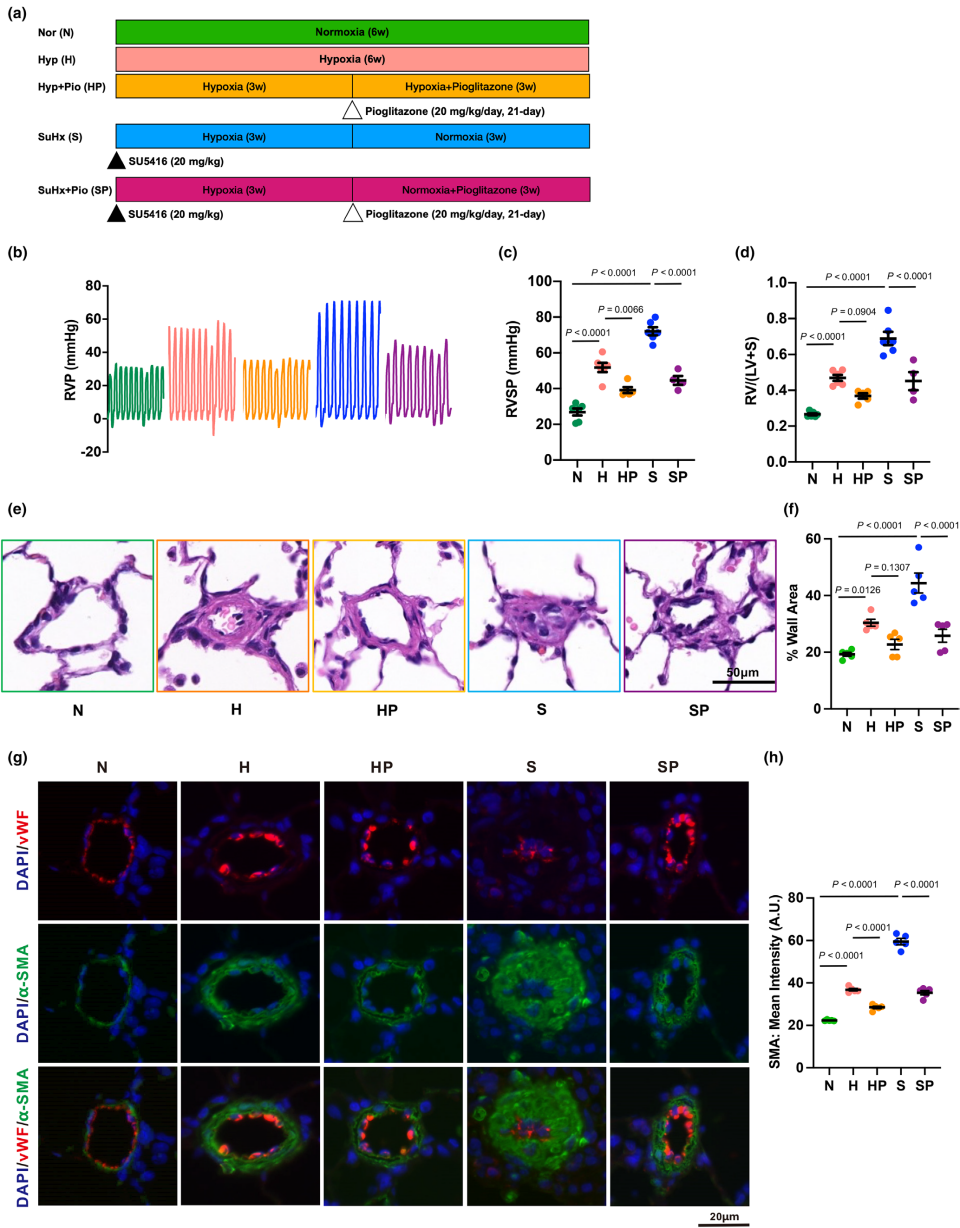
Pioglitazone inhibits experimental PH rat models induced by hypoxia or SuHx. (a) Flow chart showing the experimental design. (b–d) Trace (b) and analyzed graphs (c and d) representing the right ventricular systolic pressure (RVSP, c), and Fulton Index (d). Scatter plot values were mean ± SEM, *n* = 4–6 rats in each group. (e and f) Hematoxylin and eosin (H&E) staining images (e) and analyzed bar graph (f) of lung sections showing the thickening of the pulmonary arteries. (g and h) Representative immunofluorescent (IF) staining images (g) and analyzed bar graph (h) showing the immunofluorescent staining of both the smooth muscle cell marker alpha‐Actin (α‐SMC, green), together with DAPI (blue) which indicates the nucleus. Scatter plot values were mean ± SEM, *n* = 5 rats in each group. Scale bar indicates 50 μm (e) and 20 μm (g), respectively.

A total of five groups including Normoxia (N), Hypoxia (H), Hypoxia + Pioglitazone (HP), SuHx (S), and SuHx + Pio (SP) were designed to study the therapeutic role of pioglitazone on PH (Figure 1a). Typical PH characteristics, such as the right ventricular systolic pressure (RVSP, Figure 1b,c) and right ventricular hypertrophy (RVH) reflected by the Fulton Index (Figure 1d), were observed in “H” and “S” groups, comparing to “N”. Treatment of pioglitazone attenuated the RVSP and RVH in “HP” and “SP” groups, comparing to “H” and “S” groups (Figure 1b–d). Lung histological analysis showed obvious thickening and remodeling of the smooth muscle media wall in distal intrapulmonary vessels with an outer diameter (OD) <50 μm, indexed by the % wall area in “H” and “S” groups, which were attenuated in “HP” and “SP” groups (Figure 1e,f). Immunofluorescent double staining on the lung slides by labeling the specific markers for vascular smooth muscle (α‐SMA, green) and endothelium (vWF, red) were also performed, and the immunofluorescent intensity of α‐SMA was quantified. Similarly, treatment of pioglitazone significantly inhibited the smooth muscle thickening in “HP” and “SP” groups (Figure 1g,h).

### 3.2 Altered gut microbiome profile in HPH and SuHx‐PH rats treated with or without pioglitazone

**FIGURE 2:**
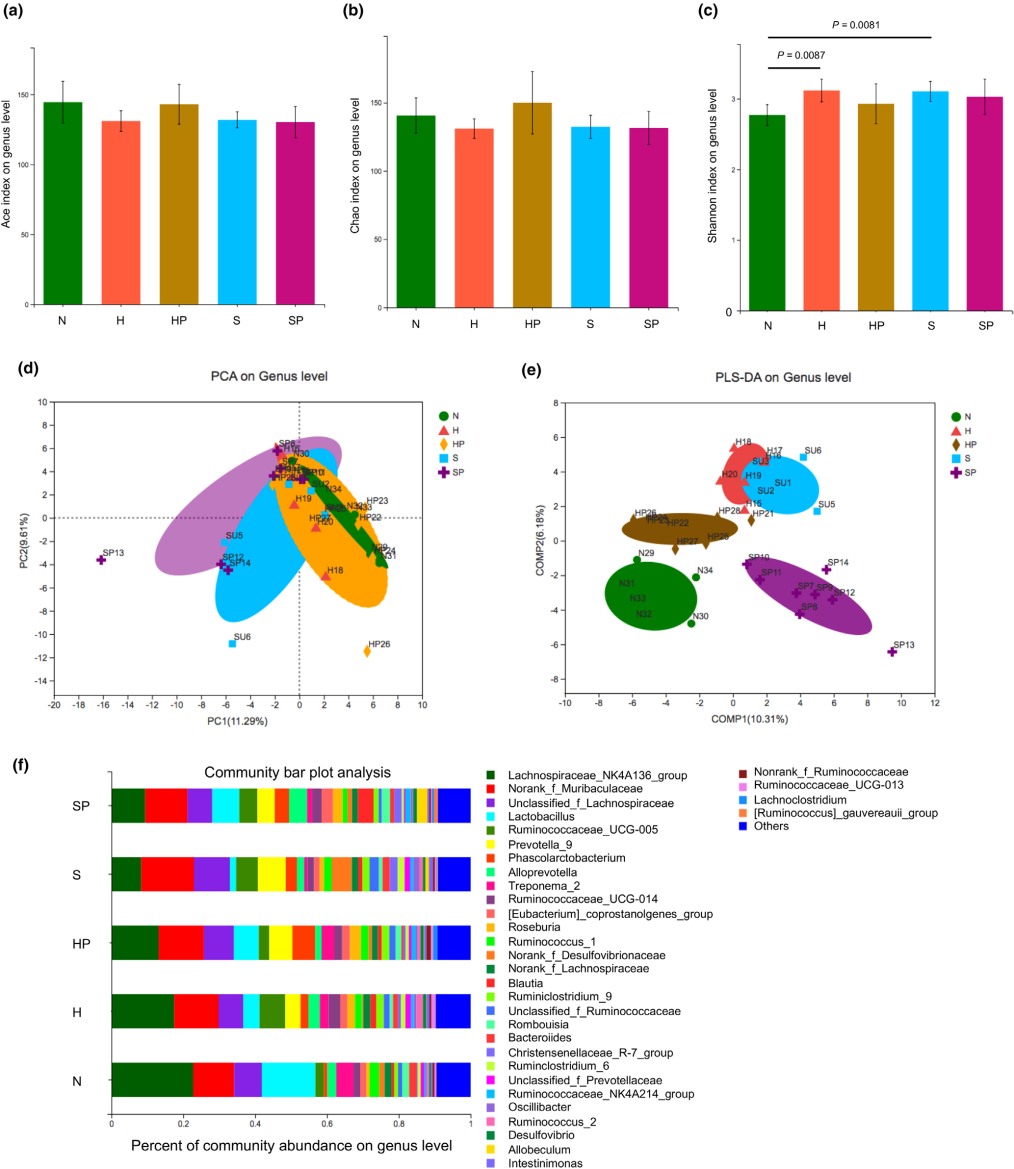
Altered gut microbiome in HPH and SuHx‐PH rats treated with or without pioglitazone. (a–c) α‐diversity indexes showing Ace (a), Chao (b), and Shannon (c) at genus level. (d and e) β‐diversity showing PCA (d) and PLS‐DA (e) of the microbiome profile. (f) Percentage bar graph showing the relative abundance of microbiota at genus level.

Firstly, α‐diversity analysis showed slight changes in the richness and diversity of gut microbiome, indexed by Ace (Figure 2a), Chao (Figure 2b), and Shannon (Figure 2c) indexes. Then, β‐diversity was also assessed by principal component analysis (PCA, Figure 2d) and partial least squares discriminant analysis (PLS‐DA, Figure 2e) to represent the clustered microbiome profile at genus level. The percentage community composition among the five groups were outlined (Figure 2f).

### 3.3 Altered plasma metabolite profile in HPH and SuHx‐PH rats treated with or without pioglitazone

**FIGURE 3:**
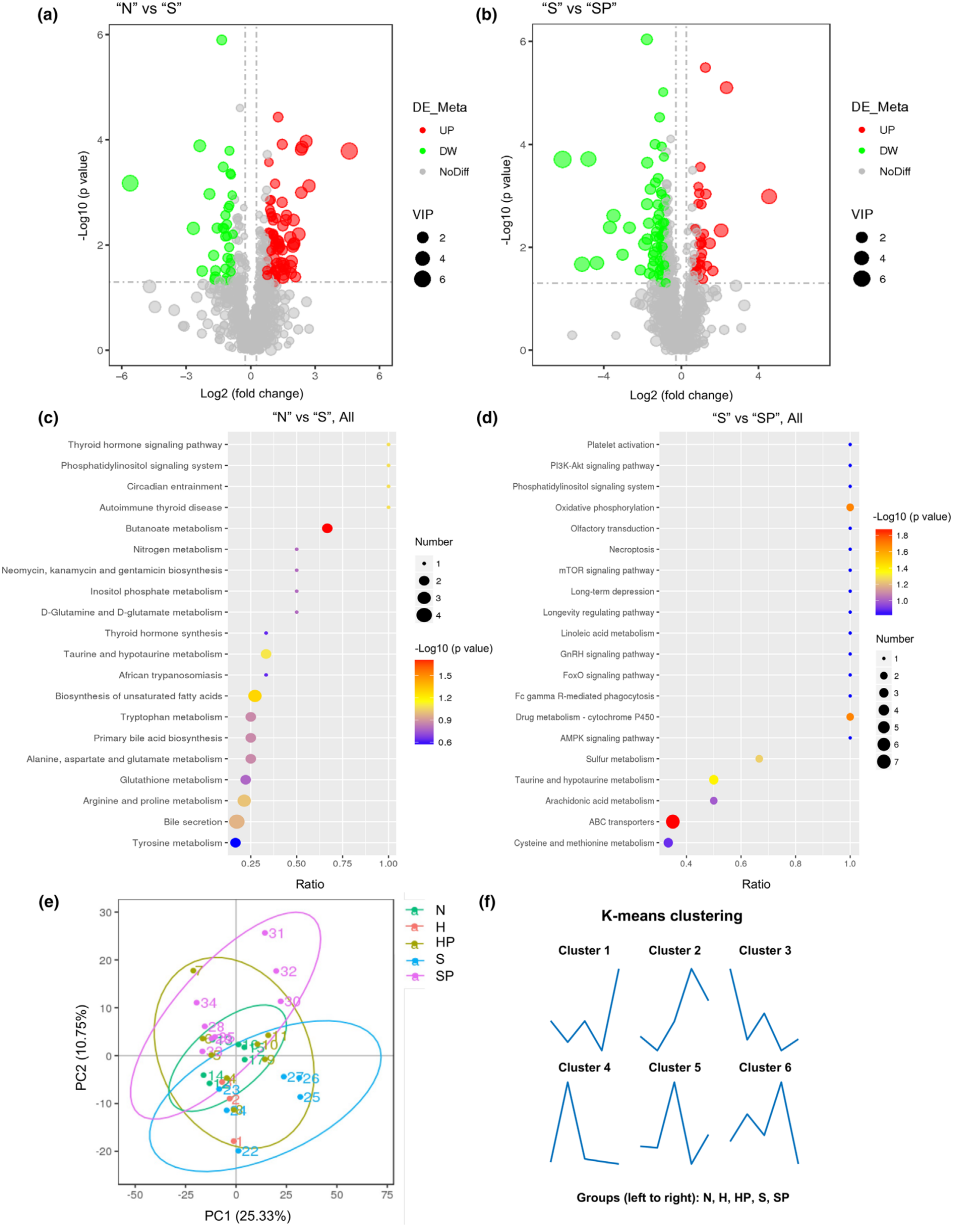
Altered plasma metabolome in HPH and SuHx‐PH rats treated with or without pioglitazone. (a and b) Volcano plots representing the plasma metabolites between “N” versus “S” (a) and “S” versus “SP” (b), where red and green indicated upregulated and downregulated groups. (c and d) KEGG pathway analysis for the identification of changed pathways between “N” versus “S” (c) and “S” versus “SP” (d). (e) β‐diversity showing PCA of the metabolome profile. (f) The k‐means clustering of metabolites.

Differences among “N”, “S”, and “SP” groups were selected for further analysis due to more significant separation. Volcano plots represented the specific up‐ and down‐regulated metabolites between “N” and “S” groups (Figure 3a, Table S1), as well between “S” and “SP” groups (Figure 3b, Table S1). For instance, increased plasma level of spermine, which has recently been shown elevated in patients with PH (He et al., 2020; Zhu et al., 2019) and contributes to the PH pathogenesis through activating calcium‐sensing receptor (CaSR) and inducing subsequent smooth muscle cell constriction and proliferation (Yamamura et al., 2012; Zhu et al., 2019), was also confirmed in the “H” and “S” groups. Then, KEGG analysis showed distinct shifts in metabolic pathways among “N”, “S”, and “SP” groups. In specific, the “Butanoate metabolism” and “ABC transporters” pathways were most significantly affected by the altered metabolite profile between “N” and “S” (Figure 3c), and between “S” and “SP” (Figure 3d). Significant separation of the metabolite profile among the five groups, reflected by the principal coordinate analysis (PCoA) analysis (Figure 3e). As described in Figure 3f,k‐means clustering was used to classify the differentially regulated plasma metabolites into six clusters. The detailed composition of metabolites in six clusters were listed in Table S2.

### 3.4 Correlation analysis between different groups of gut microbiome taxa and plasma metabolite clusters

**FIGURE 4:**
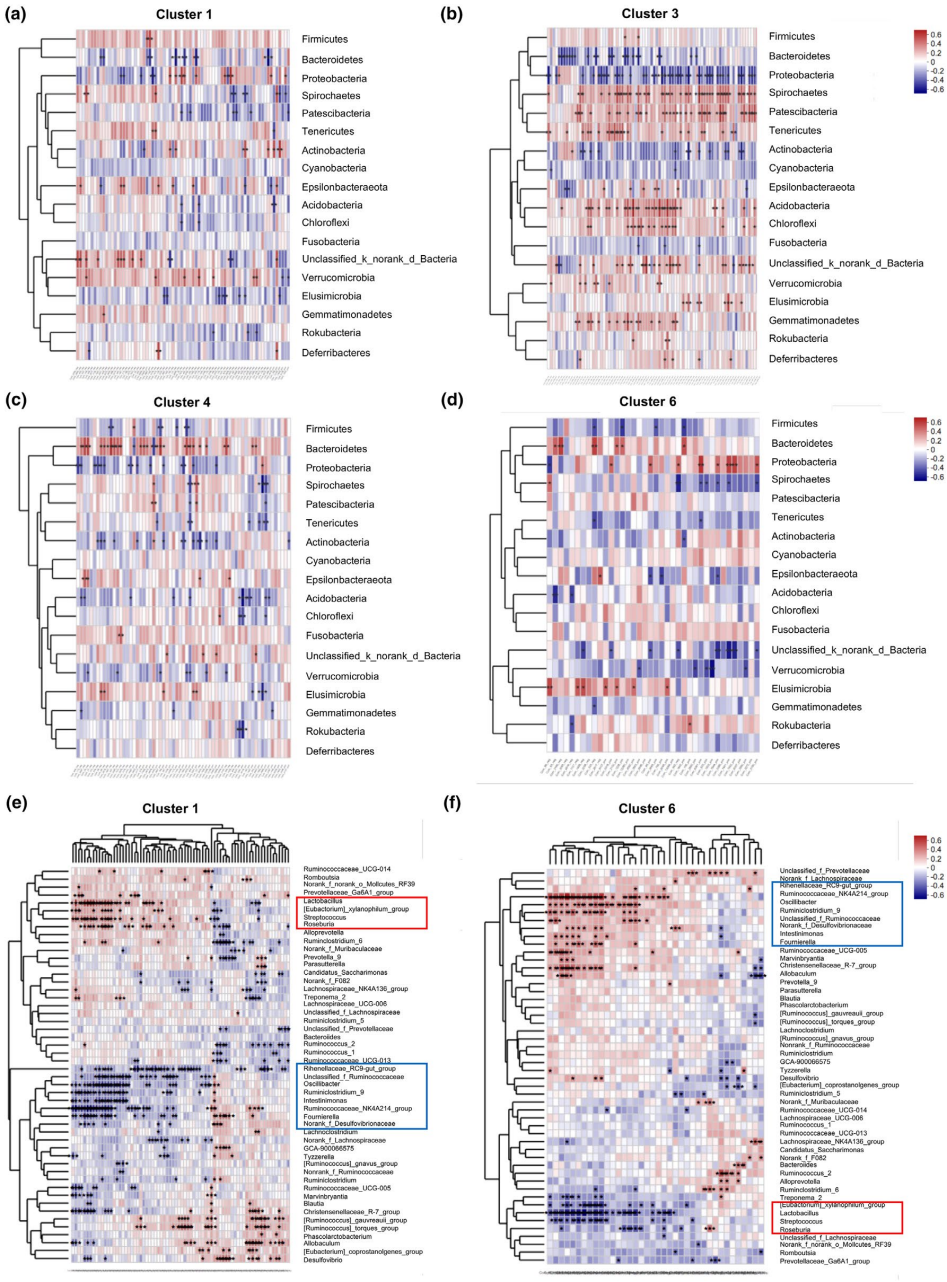
Correlation analysis between gut microbiome and plasma metabolome. (a–d) Heatmaps of the Spearman's correlation coefficients between changes in Cluster 1 (a), Cluster 3 (b), Cluster 4 (c), and Cluster 6 (d) of metabolites and bacteria phyla caused by PH circumstances and pioglitazone intervention. (e and f) Heatmaps of the Spearman's correlation coefficients between changes in Cluster 1 (e) and Cluster 6 (f) of metabolites and bacteria genera caused by PH circumstances and pioglitazone intervention. The data were adjusted for body weight, fat mass, and waist‐to‐hip ratio. ^+^ *p* < 0.05, *FDR <0.1, and **FDR <0.05 as indicated. Pink and blue in the far‐right column indicated increased and decreased relative abundance, respectively. Only taxa with significant correlations (at least one based on FDR or two based on raw *p* value) were marked.

Spearman's correlation analysis was performed to further dissect the potential correlation between the different clusters of metabolites and the gut microbiota. As shown in Figure 4a–d, the major phyla represented divergent correlation with different clusters of metabolites. For example, the major phyla *Firmicutes* and *Bacteroidetes* showed almost completely divergent correlation pattern with Group 1, including Cluster 1 (Figure 4a) and Cluster 3 (Figure 4b), and Group 2 metabolites, including Cluster 4 (Figure 4c) and Cluster 6 (Figure 4d). We also uncovered strong, unique association between the plasma metabolites and the gut microbiome at genus level. *Roseburia*, *Lactobacillus*, *Streptococcaceae*, and [*Eubactorium*]*_xylanophilum_group* (red box) positively correlated with the Cluster 1 (Figure 4e), whereas negatively correlated with the Cluster 6 (Figure 4f). According to previous publications, these genera have been shown with beneficial consequences, such as butyrate‐producing and immune balancing, etc. (Zimmermann et al., 2019). In contrast, *Rihenellaceae_ RC9‐gut_group*, *Norank_f_Desulfovlbrionaceae*, *Ruminococcaceae_NK4A214_group*, *Oscillibacter*, *Ruminiclostridium_9*, *Unclassified_f_Ruminococcaceae*, *Intestinimonas*, and *Fournierella* (blue box) positively correlated with the Cluster 6 (Figure 4f), whereas negatively correlated with the Cluster 1 (Figure 4e). These bacteria genera have been shown to be associated with many chronic lung diseases and heart diseases (Gutiérrez‐Calabrés et al., 2020; Paudel et al., 2020).

### 3.5 Supplementation of *R. intestinalis* partially attenuates the pathogenesis in SuHx‐PH rats

**FIGURE 5:**
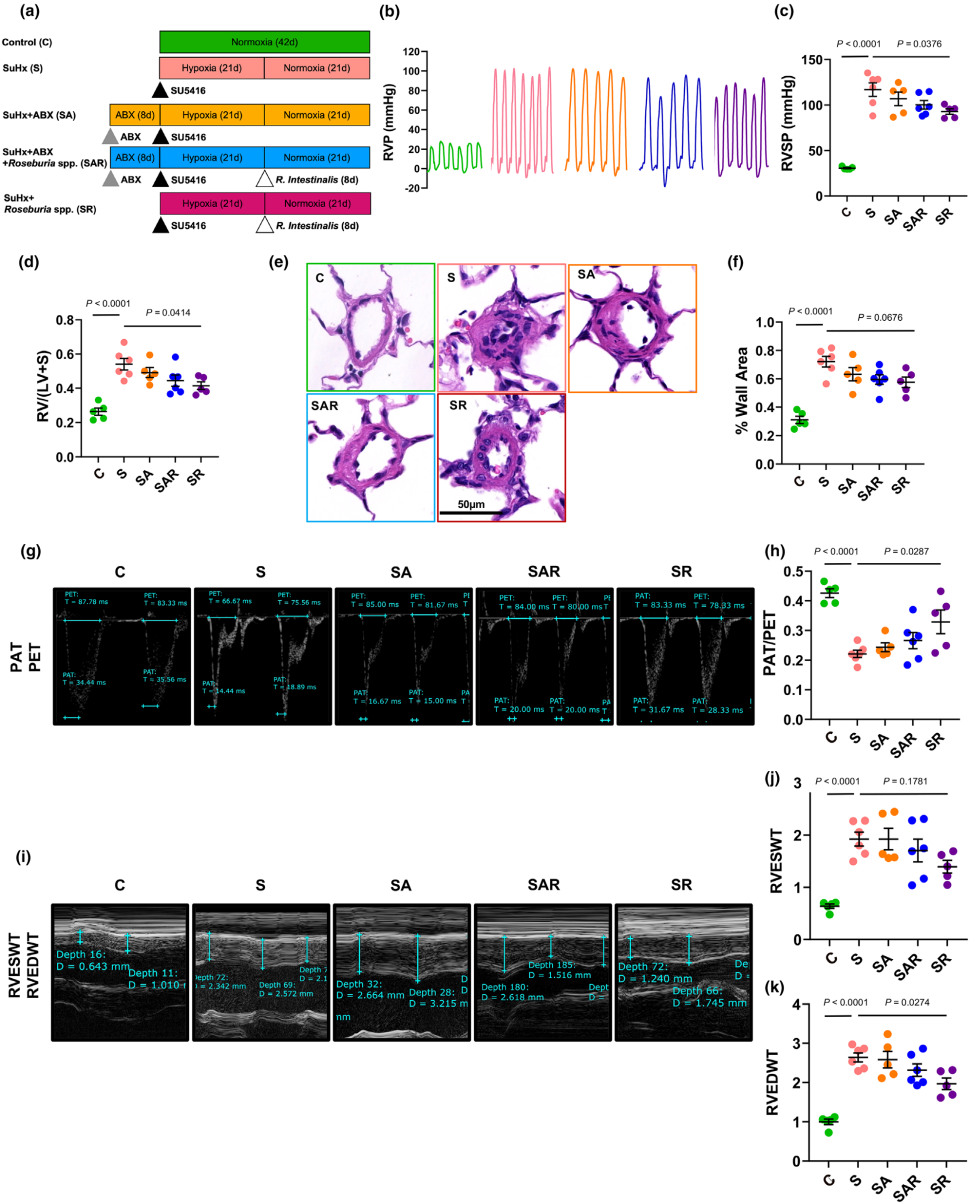
Supplementation of *R. intestinalis* effectively attenuates the pathogenesis in SuHx‐PH rats. (a) Flow chart showing the experimental design. (b–d) Trace (b) and analyzed graphs (c and d) representing the RVSP (c) and Fulton Index (d). Scatter plot values were mean ± SEM, *n* = 5–6 rats in each group. (e and f) H&E staining images (e) and analyzed bar graph (f) of lung sections showing the thickening of the pulmonary arteries, *n* = 5 rats in each group. Scale bar indicates 50 μm. (g–k) Echocardiography (g and i) and analyzed bar graph (h, j, and k) showing the indexes of right heart function, PAT/PET, RVEDWT, and RVESWT. Scatter plot values were mean ± SEM, *n* = 5–6 rats in each group.

A total of five groups, including Normoxia control (C), SuHx (S), SuHx + Antibiotics (SA), SuHx + Antibiotics + *R. intestinalis* (SAR), and SuHx + *R. intestinalis* (SR), were designed to assess the role of *R. intestinalis* on PH pathogenesis (Figure 5a). Supplementation of *R. intestinalis* partially inhibited the RVSP and RVH (Figure 5b–d), and the thickening of distal intrapulmonary vessels (Figure 5e,f), and the right heart function indexes, including the PAT/PET, RVEDWT, and RVESWT, reflected by the echocardiography (Figure 5g–k) in the “SR” group. However, the role of *R. intestinalis* is not sufficient in the “SAR” group, probably due to the resident effects of antibiotics on bacterial implantation and the relatively short period of bacterial supplementation (8 days).

### 3.6 Supplementation of *R. intestinalis* reshapes the gut microbiome in SuHx‐PH rats

**FIGURE 6:**
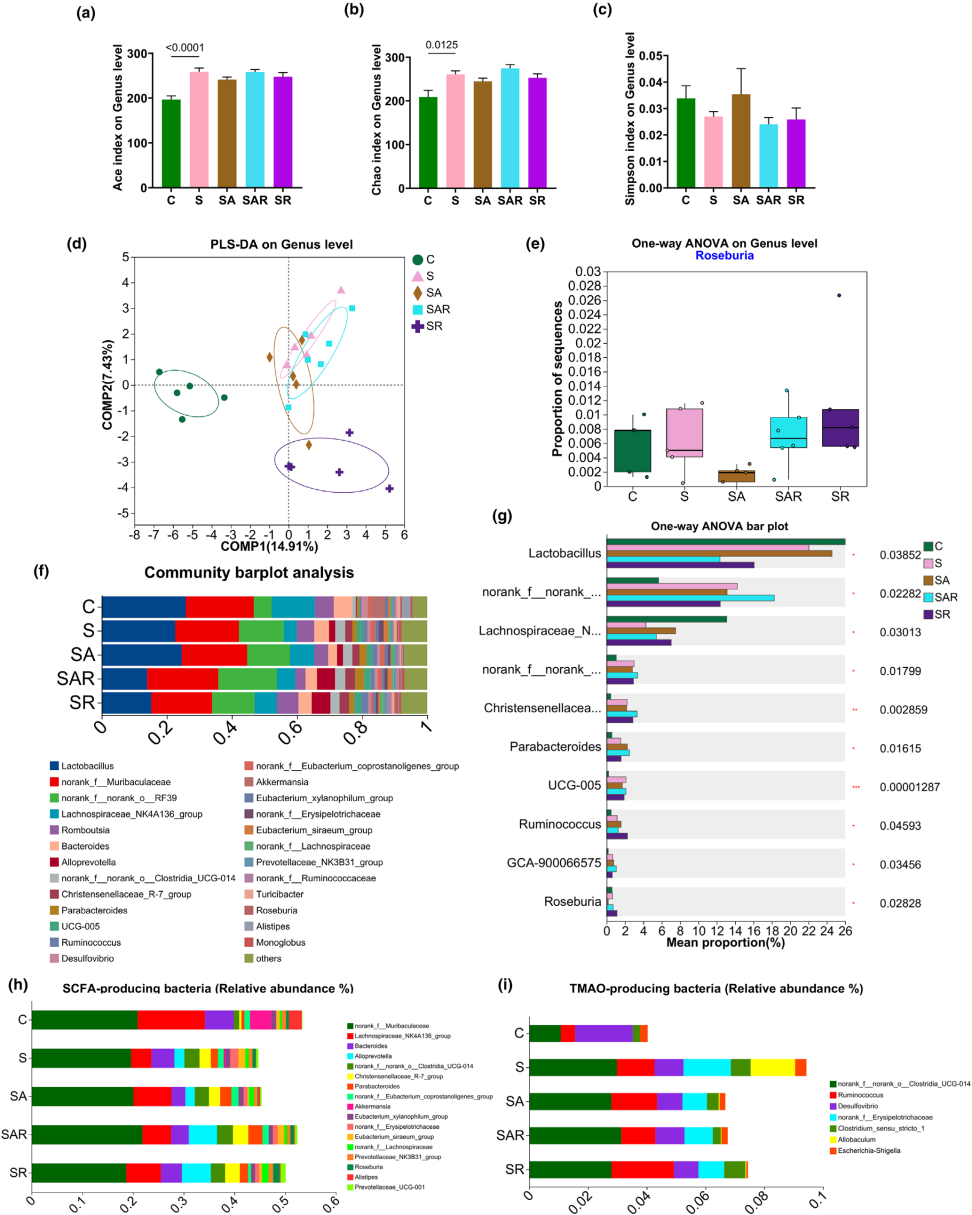
Supplementation of *R. intestinalis* reshapes the gut microbiome in SuHx‐PH rats. (a–c) α‐diversity indexes showing Ace (a), Chao (b), and Simpson (c) at genus level. (d) β‐diversity showing the PLS‐DA of microbiome profile, *n* = 5–6 rats in each group. (e) Bar plot showing the relative abundance of *Roseburia*. (f) Percentage bar graph showing the relative abundance of microbiota at genus level. (g) Kruskal–Wallis H test bar plot representing the top fifteen mostly altered taxa among five groups; *p* values were listed as indicated. (h and i) Percentage bar graph showing the relative abundance of SCFA‐producing bacteria (h) and TMAO‐producing bacteria (i).

First, α‐diversity analysis showed slight changes in the richness and diversity of gut microbiome, indexed by Ace (Figure 6a), Chao (Figure 6b), and Simpson (Figure 6c) indexes. After supplementation of *R. intestinalis*, PLS‐DA indicated separation of the microbiome profile at genus level (Figure 6d). To validate the efficiency of *R. intestinalis* supplementation, the abundance of *Roseburia* was increased in “SR” versus “S” and in “SAR” versus “SA” (Figure 6e). Analysis on the microbial community at phylum level represented specific changes in selective top abundant genera (Figure 6f,g) among the five groups. Previous studies have reported the elevated abundance of trimethylamine N‐oxide (TMAO)‐producing bacteria (Huang et al., 2022; Yang et al., 2022) and reduced abundance of short‐chain fatty acids (SCFA)‐producing bacteria (Karoor et al., 2021; Moutsoglou et al., 2023) are associated with the disease development of PH; we then analyzed and uncovered elevated abundance of TMAO‐producing bacteria and reduced SCFA‐producing bacteria in the “S” group, which were partially recovered in the “SR” and “SAR” groups (Figure 6h,i).

### 3.7 Supplementation of *R. intestinalis* rebuilds the plasma metabolome in SuHx‐PH rats

**FIGURE 7:**
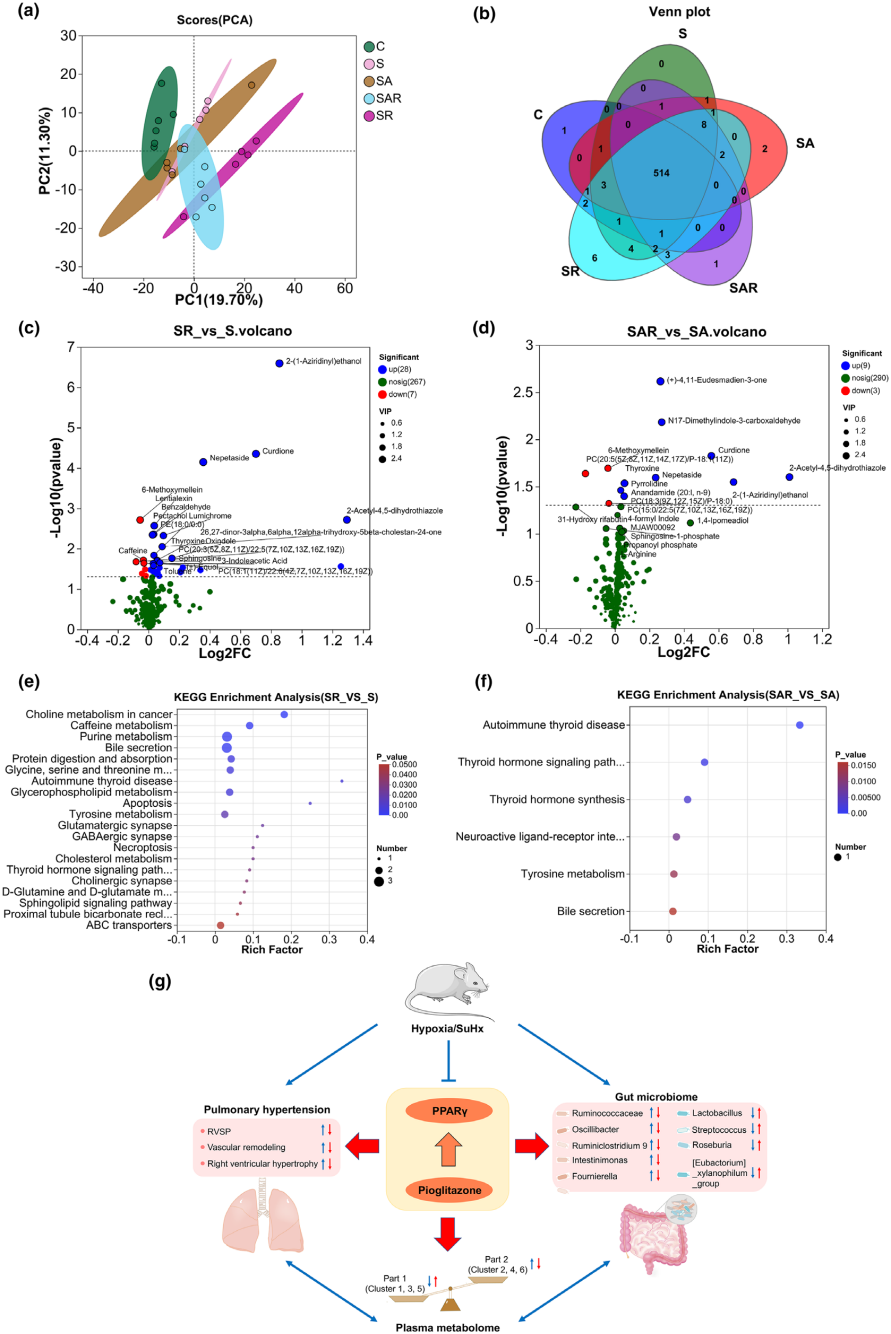
Supplementation of *R. intestinalis* rebuilds the host metabolome in SuHx‐PH rats. (a) Differences in β‐diversity were measured by PCA representing grouped microbiome profile, *n* = 5–6 rats in each group. (b) Venn diagram showing the shared and unique plasma metabolites in each group. (c and d) Volcano plots representing the plasma metabolites between “SR” versus “S” (c) and “SAR” versus “SA” (d). (e and f) KEGG enrichment analysis showing the enriched pathways in “SR” versus “S” (e) and “SAR” versus “SA” (f). (g) Proposed working model of the study.

After supplementation of *R. intestinalis*, PCA indicated separation of the microbiome profile at genus level (Figure 7a). Although majority of the metabolites were consistently shown in all five groups, unique metabolites were also captured in specific group (Figure 7b). Volcano plots represented the specific up‐ and down‐regulated metabolites between “S” and “SR” groups (Figure 7c), and between “SA” and “SAR” groups (Figure 7d). Notably, consistent enriched metabolites, such as curdione, which has been reported to be beneficial in pulmonary fibrosis (Liu et al., 2020) and sepsis‐induced lung injury (Yang et al., 2023), as well as nepetaside, 2‐acetyl‐4,5‐dihydrothiazole, and 2‐(1‐aziridinyl)ethanol, exerting anti‐inflammation effects, in “SR” and “SAR” groups (Figure 7c,d). Dramatic alteration was seen in the plasma metabolome profile in “SR” than “SAR” group, in line with the higher efficiency of “SR” than “SAR” group on PH pathogenesis. KEGG analysis uncovered enriched pathways including “purine metabolism”, “bile secretion”, “choline metabolism,” and “caffeine metabolism” in “SR” versus “S” group (Figure 7e,f).

## 4 DISCUSSION

In this study, we analyzed and uncovered unique correlations among the PH characteristics, gut microbiome, and plasma metabolome, suggesting novel mechanisms underlying the therapeutic action of PPARγ agonist pioglitazone. Supplementation of specific enriched probiotic bacteria, such as *R. intestinalis*, functionally attenuated the SuHx‐PH, providing a new strategy for the treatment of PH based on modulation of gut microbiome.

Bar et al. reported that comparing to host genetics and clinical parameters, diet and microbiome had stronger predictive power of metabolites variance in human subjects (Bar et al., 2020), suggesting the significance of altered microbiome on host metabolome and diseases. Identifying gut microbes that affect the human health and systematically correlating the altered microbiome, metabolome, and disease pathogenesis will be of importance for early prevention and personalized treatment of human diseases. From food sources, the microbial community produces metabolites such as SCFAs and TMAO that affect a range of metabolic factors, control adiposity, and predispose individuals to many diseases, including PH (Koeth et al., 2013; Zhang et al., 2021). Application of medications, especially orally used drugs, acts as another important factor, with drugs such as antibiotics, proton‐pump inhibitors, metformin, and antidepressants have been shown to dramatically shift the microbiota profile, in most cases to a less diverse composition (Zhernakova et al., 2016). The gut microbiome is strongly linked with intrinsic factors such as host metabolic and immune status. It has been estimated that variations in gut microbiome can explain up to 6% of the variation in blood lipid levels in healthy individuals (Fu et al., 2015). In this study, using SPF‐grade rat models, which were housed in the same circumstance and fed with equal background of dietary, unique, divergent profile changes of gut microbiome have been uncovered under circumstances of PH and PPARγ activation by oral administration of pioglitazone. Previous studies have reported the microbial trends at family and genus levels are more stable and better biomarkers for indexing PH. Sharma et al. reported increased abundance of *Prevotella*, *Oscillospira*, and *Ruminococcus*, and decreased abundance of *Lactobacillus* in HPH mice versus normoxic control mice (Sharma, Oliveira, Yang, Karas, et al., 2020), mostly in line with our findings in HPH and SuHx‐PH rats versus normoxic control rats. Moreover, the altered microbiota composition can be mostly normalized by pioglitazone treatment.

According to previous research, gut bacteria, such as the *Prevotella copri* and *Bacteroides vulgatus*, are associated with insulin resistance in humans and induce insulin resistance in mice (Pedersen et al., 2016). Notably, Zhao et al. reported that excessive intracolonic saturated long‐chain fatty acids (SLCFAs) are associated with enhanced bowel motility in neonatal maternal separation rats, and increased SLCFAs positively correlated with elevated abundances of *Prevotella*, *Lactobacillus*, and *Alistipes* (Zhao et al., 2018). In our study, the abundance of *Prevotella* was significantly increased in “H” and “S” groups versus control group, while decreased in “SP” group. In addition, *Roseburia* was part of commensal bacteria that produce SCFAs, especially butyrate, which exert anti‐inflammatory properties and improve colonic motility (Arpaia et al., 2013). Moreover, the combined risk score in patients with inflammatory bowel disease has also been shown to be associated with a decreased abundance of *Roseburia* (Imhann et al., 2018). The family *Ruminococcaecae* are obligate anaerobes that can induce gut dysbiosis and inflammation of airway, lung and colon in mice (Chua et al., 2018). In a large‐scale cohort study, *Roseburia* spp., *Clostridium* spp., *Romboutsia* spp., and *Ruminococcaceae* spp. have been screened as the best predictors for systolic blood pressure (Verhaar et al., 2020). In addition, strong association has been reported between increased gut genera *Oscillospira*, *Prevotella 9* and *Ruminiclostridium 6* and heart failure. As reviewed by Jin et al., increased abundance of host opportunistic pathogens (such as *Escherichia coli*, *Clostridium ramosum*, *Bacteroides caccae*, and *Eggerthella lenta*) and reduced abundance of SCFA‐producing bacteria (such as *Roseburia*, *Faecalibacterium*, and *Eubacterium rectale*) have been associated with increased risks of cardiovascular diseases (Jin et al., 2021). Although the concept using healthy microbiota transplantation to treat PH has been proposed for several years (Moutsoglou, 2022; Moutsoglou et al., 2023), majority of the current studies in this field are still based on a description on abundance/profile level. Data in this study first proved that supplementation of *R. intestinalis* partially attenuates the disease development of SuHx‐PH rat model, providing direct experimental evidence for future designing and optimizing novel anti‐PH treatment strategies based on the regulation of host microbiota.

As summarized in Figure 7g, this study uncovered the diversity of gut microbiota and its metabolism as key determinants for the heterogeneous adaptation to PH circumstances and orally pioglitazone intervention, demonstrating that: (1) the reorganization of gut microbiota composition may drive either “pathogenic” or “therapeutic” consequences; (2) distinct enrichment signatures of the gut microbiome were observed in PH model and pioglitazone‐treated rats, which represented strong association with the PH pathogenic and pioglitazone therapeutic outcomes; (3) manipulation of the gut microbiome by supplementation of “probiotics” (such as *R. intestinalis*) could be natural, healthy and safe strategy for the prevention, adjuvant therapy and/or treatment of PH, leading a new avenue that deserves further evaluation. However, how pioglitazone imposes such divergent impacts on the composition and function of gut microbiota warrants further investigation. PH is associated with impaired gut microenvironments, such as the imbalanced inflammatory and oxidative status, all contribute to altered gut microbiota composition. Pioglitazone may directly amplify the subtle differences of the gut microenvironments, reshape the gut microbial diversity, and therefore benefit PH. Since the variations in gut microbiome may deeply impact the variation in blood lipid levels, we assume the altered gut microbiome triggers, at least partly, the profile changes of metabolome and ultimately leads to consequences on PH. In addition, PPARγ can also be activated by metabolites, such as the microbial‐produced butyrate, which in turn retrieves the dysbiosis in rats (Byndloss et al., 2017). These mechanisms raise the possibility to render a positive feedback loop to exert anti‐PH consequences. This study provided translational implications into the mechanisms of PPARγ agonist pioglitazone for PH therapy and also implied that orally administrated medications may significantly reshape gut microbiome and host metabolome, bringing up a new direction for PH research that has not been previously elucidated.

## AUTHOR CONTRIBUTIONS

K.Y. initiated and designed the project, wrote and edited the manuscript; Z.Z. and S.M. performed the animal experiments, performed the bioinformatics analysis, analyzed the data, and prepared the figures; Y.R.L. and M.Z. initiated and designed the project, performed the bioinformatics analysis, analyzed the data, prepared the figures, wrote and edited the manuscript; C.Z., Y.L., X.S., and C.W. performed the animal experiments; S.L., J.L., X.Y.L., and J.Z. contributed to the sample collection and 16S rRNA sequencing experiments; C.H., Y.C., N.L., L.X., Q.J., and W.H. contributed to the sample collection and the plasma metabolome experiments; J.W. and W.L. provided constructive consultant and advise of the project; Z.W. provided constructive consultant and advise of the project, and edited the manuscript; All authors approved the final submission of the paper.

## FUNDING INFORMATION

This work was supported in part by the National Natural Science Foundation of China (82170065, 82241012, 82270052, 82120108001, 82370063, 82170069), Open Research Funds from The Sixth Affiliated Hospital of Guangzhou Medical University (Qingyuan People's Hospital) (202201–309, 202201–101), National Key R&D Program of China (2022YFE0131500), R&D Program of Guangzhou National Laboratory (GZNL2023A02013), Local Innovative and Research Teams Project of Guangdong Pearl River Talents Program (2017BT01S155), Natural Science Foundation of Guangdong Province, China (2024A1515011208, 2024A1515013104, 2022A1515012564, 2022A1515012052), Independent Project of State Key Laboratory of Respiratory Disease (SKLRD‐Z‐202219, SKLRD‐Z‐202207, SKLRD‐Z‐202408), and Plan on Enhancing Scientific Research in Guangzhou Medical University.

## CONFLICT OF INTEREST STATEMENT

The authors declare none competing interests.

## ETHICS STATEMENT

The animal protocols were approved and conducted by the Animal Care and Use Committee of the First Affiliated Hospital of Guangzhou Medical University (ethical approval number: 2018‐456).

## Supporting information


Table S1.

Table S2.

## Data Availability

The data that support the findings of this study are available from the corresponding authors upon reasonable request.
